# Correction: Active versus sham transcranial direct current stimulation (tDCS) as an adjunct to varenicline treatment for smoking cessation: Study protocol for a double-blind single dummy randomized controlled trial

**DOI:** 10.1371/journal.pone.0318721

**Published:** 2025-01-30

**Authors:** Laurie Zawertailo, Helena Zhang, Noreen Rahmani, Tarek K. Rajji, Peter Selby

This acknowledgement was excluded from the published manuscript. The correct Acknowledgement section is as follows: The authors wish to acknowledge the in-kind contribution of transcranial direct current stimulation (tDCS) devices and support from Nuraleve Inc., Ottawa, Canada.

The following information is missing from the Competing Interests section: tDCS devices and device support are provided by Nuraleve Inc., Ottawa, Canada. Nuraleve has no input into the study design, conduct, or analysis and interpretation of the results. They have no role in the writing or approval of the manuscript.
