# Supplementary material for: Active versus sham transcranial direct current stimulation (tDCS) as an adjunct to varenicline treatment for smoking cessation: Study protocol for a double-blind single dummy randomized controlled trial
Source: PLoS One. 2022 Dec 8;17(12):e0277408. doi: 10.1371/journal.pone.0277408 (PMC9731486; doi:10.1371/journal.pone.0277408)
Supplement: S2 Appendix — Copy of the informed consent form. (PDF) [file pone.0277408.s002.pdf]

Appendix S2. Study Consent Form

Study Information Sheet

Using non-invasive brain stimulation (tDCS) to improve the effectiveness of varenicline for treating tobacco dependence: a randomized controlled trial

Principal Investigator: Laurie Zawertailo, PhD

Co-Investigators: Peter Selby, MBBS (Qualified Investigator)
Martin Zack, PhD (Co-Investigator)
Tarek Rajii, MD, PhD (Co-Investigator)

Study Site: Nicotine Dependence Clinic
Centre for Addiction & Mental Health (CAMH)
175 College Street, Toronto Ontario

Purpose:

This research will test the effect of a standard treatment for smoking cessation (varenicline or Champix) combined with a procedure called trans-cranial direct current stimulation (tDCS). tDCS is not a standard treatment for quitting smoking. However, it has shown some promising effects in laboratory experiments with smokers who were not trying to quit. This research will test whether the addition of tDCS can increase your ability to quit smoking when combined with medication. In addition, this research study involves brain scans using functional magnetic resonance imaging (fMRI) before the start of treatment and again at the end of treatment. The purpose of these scans is to determine if the tDCS procedure changes your brains' response to smoking cues and reward anticipation. Lastly, visual scanning tests will be conducted periodically within the first 12 weeks to improve our understanding of tDCS and changes in brain function.

Study Procedure:

1. Study Visits. Participation involves coming to the Nicotine Dependence Clinic of CAMH located at 175 College Street 20 separate times: one pre-study assessment visit; 3 fMRI scan sessions; 10 consecutive daily tDCS treatment sessions (scheduled Monday to Friday); 5 tDCS booster sessions; and one in-person follow up visit 3 months after completing the study. These are described in detail below. You will receive 2 transit tokens to cover round-trip fare to CAMH for all study visits.

    a. Pre-Study Assessment. A number of test and procedures will be completed in order to assess your suitability to participate in this study. This 3 hour session will involve:

        i. *Brief Physical Screening*: A study coordinator will measure your heart rate, weight, and blood pressure. You will also be asked to complete 2 breath tests to determine (i) the absence of alcohol in your system, and (ii) the level of carbon monoxide in your system, a by-product of smoking. You will also be asked to provide a urine sample to test for drugs or medications that may affect your response to tDCS, and to exclude pregnancy.

        ii. *Clinical Interview*: A clinical interview will be used to determine whether you may be suffering from mental health and/or addiction symptoms, aside from nicotine dependence.

iii. *Questionnaires*: You will complete computer-based questionnaires regarding your smoking habits, use of alcohol and drugs, mood, and personality.

iv. *Cognitive Task*: You will complete a 20 minute computer-based task that measures attention and reaction time.

b. tDCS Sessions: If you are eligible based on your assessment, you will be randomly assigned to one of two tDCS treatment groups: the Active Group (actual tDCS stimulation) or the Inactive Group (the stimulator will be applied and turned on but won't be active). Neither you nor the researcher conducting the study will know which group you have been assigned to. You have an equal chance of being assigned to the Active Group or the Inactive Group. You will also receive the medication varenicline (Champix) to take every day as prescribed for 12 weeks. You will be required to attend 10 daily tDCS sessions (Monday through Friday for 2 consecutive weeks) plus 'booster sessions' every 2 weeks over the remaining 10 weeks of treatment. Each session will be the same in terms of the things you will be asked to do. At the beginning of each session you will undergo a physical screening as above, excluding the contribution of a urine sample, but adding a brief scalp exam and temperature reading. Then you will complete some questionnaires. Under the supervision of a facilitator, you will receive 20 minutes of tDCS, during which you will read assigned materials. You will be videotaped during each session to help standardize the treatment you will be getting, and for your safety. The session will end with the completion of a few questionnaires. These sessions will each take less than one hour to complete.

In order to prevent potentially dangerous interactions with tDCS, you will be required to refrain from alcohol for 12 hours before the start of each session. You will also be required to abstain from all illicit mood-altering drugs, and all holistic and herbal preparations during the 12 week duration of this study.

c. Functional magnetic resonance imaging (fMRI) scan sessions: You will undergo fMRI scans up to three separate times: twice prior to starting the study treatment and once at the end of the 12 week treatment period. fMRI is a non-invasive procedure that measures your brain's activity in response to various picture cues and there are very few reported side effects. The MRI machine looks like a big doughnut, and you will lie down on a bed with your head and shoulders in the tunnel made by the "doughnut hole". We will put some pillows around your head to keep it from moving and then ask you to stay very still while we scan your brain to get the pictures. You should try to remain as still as possible during the scans. Movements will not be dangerous to you in any way, but will blur the picture of your brain. For each MRI session (two before you start the study treatment and one when you complete the study treatment), you will need to hold still in the machine for approximately one hour. The MR technologist will be able to observe you at all times. You will be able to contact the MR technologist at any time during the scan session for any reason. You will hear moderately loud knocking or beeping sounds when the MRI machine is scanning. You will be given ear protection to wear in the scanner. Different types of scans will make different types of sounds, which is normal for MRI. The technologist will talk to you before each scan starts. There will be a mixture of very short scans and some longer scans (up to 13 minutes each).

d. Visual Scanning Tests: One way to study how the brain works is to examine how the eyes respond to visual stimuli. This study will explore whether eye responses are different in smokers using varenicline vs. smokers using varenicline and tDCS when looking at pictures on a computer screen. You will be performing visual scanning tests on 5 occasions during the study, which will generally take around 20 minutes each time to complete. You will be doing the same test each session. During these tests, you will be asked to view a series of images on a laptop screen of

various content: desks, pens, cigarettes, people smoking cigarettes etc. The laptop will be equipped with a camera that will measure your gaze when looking at these pictures.

e. Follow-up: As part of our standard follow-up to this study, every time you come in for a visit, you will be asked to complete a few questionnaires. This will occur every weekday for the first 2 weeks (for the consecutive tDCS sessions) and then every other week during the boost sessions (5 total) and one during your final in-person visit 3 months after the end of treatment. These will take around 30 minutes to complete.

Withdrawal from the Study:

a) You are free to drop out of the study for any reason at any time. Failure to complete the study in no way will affect your current or future treatment at CAMH.

b) The study investigator/facilitator is free to stop your participation before the study is over if you do not follow the study requirements. In this case, you would receive the payment earned for your participation up to that point (see Payment Schedule attached). You will be paid at the end of each completed study visit.

Risks:

Trans-cranial Direct Current Stimulation (tDCS)
tDCS has been used in many studies with healthy volunteers and people with a variety of ailments ranging from stroke, and depression to chronic pain. In a review of 567 tDCS sessions, most of which used very similar stimulation procedures to those used in this study (2 milli-Amperes for 20-min) mild tingling of the scalp was the most common side effect (71%), followed by moderate fatigue (35%) and slight itching at electrode sites (30%). No severe adverse effects were reported. Of the more unpleasant effects, headache was most common (11%), followed by nausea (3%).

Varenicline
Using varenicline (Champix) when quitting smoking is approved by Health Canada. There are both risks and benefits of participating in this study. The risk is that there are some possible side effects of varenicline. The most common side effects of varenicline are nausea, abnormal dreams, constipation, flatulence and vomiting in 30, 13, 8, 6 and 5% of users, respectively. They are reversible and usually not severe. You may have heard in the media that some people taking varenicline have experienced severe psychiatric symptoms. These symptoms have not been proven to be caused by varenicline, but Health Canada has endorsed a public announcement about this issue that we ask you to read carefully (you may find this announcement at http://www.hc-sc.gc.ca/dhp-mps/medeff/advisories-avis/public/_2008/champix_pc-cp-eng.php).http://www.hc-sc.gc.ca/dhp-mps/medeff/advisories-avis/public/_2008/champix_pc-cp-eng.php). It is also attached to this consent form.

It is not known whether combining varenicline with tDCS will increase either the incidence or the severity of adverse effects, especially those that are common between the two treatments such as nausea and headache. You will be carefully monitored for these side effects.

fMRI
Before you can participate in an MRI study, we need to make sure it is safe for you to do so. Because certain metal objects may lead to injuries during the MRI procedure, we will ask you to answer questions about any metal implants or objects you might have in your body and the location of any tattoos. If you have any metal implants or objects that are not safe for the 3.0T MRI at CAMH, you will not be allowed to be scanned. Some objects that are not safe for MRI include cardiac pacemakers, metal fragments in the eye, aneurysm clips in your brain. If there is a strong chance you may have metal fragments in your eyes, you will need to provide an x-ray

report of your eyes before you can be scanned. The research study staff and the MR technologist will work together to make sure you will be safe in the scanner. We will also ask whether you are extremely uncomfortable in enclosed spaces (claustrophobia). Based on the use of MRI in medicine for over 20 years, most experts believe there are no long-term negative health effects caused by the magnetic field strength used in this study.
Other risks: Some people may feel uncomfortable lying still in the confined space of the MRI scanner, tingling sensations are felt by some people during certain scans or you may feel dizzy for a few minutes at the end of the MRI study. These are infrequent, but expected sensations. It is important you understand that you will be able to contact the technologist at any time during the scan. You may ask to be taken out of the scanner for any reason without any penalty or consequences.
Unexpected findings: The possibility of unexpected or incidental findings carries with it some risks. Research scans are not designed to be used for diagnosis. In the unlikely event an atypical finding is seen on your MRI scan, we may ask a radiologist or other qualified health professional to look at your scan. By signing this consent form, you agree to allow us to release the scan for review of any unexpected findings. Your identity will not be revealed. If the qualified professional recommends further tests to determine the nature and significance of any incidental findings on your MRI scan, we will contact you to help you arrange medical follow-up.
Pregnancy: Pregnant women are not candidates for research MRI studies. As with medications and other imaging procedures, it is considered prudent not to undergo MRI during pregnancy unless there is a medical need. If you are a woman of child-bearing age, we will ask you to confirm that you are not pregnant, nor likely to be pregnant, at the time of the study and prior to each scan.

Visual Scanning Tests: There are no direct risks from these tests.

Benefits:

tDCS: tDCS has been found to improve mood, and two initial studies have found that it temporarily reduces cigarette cravings.

Varenicline: Varenicline is the most effective approved treatment for quitting smoking. All study participants will be given 12-weeks of this medication at no cost as a stop smoking aid.

fMRI: There are no direct benefits to you, however the data you provide will be very important for furthering treatment for tobacco dependence.

Visual Scanning Tests: There are no direct benefits to you, however the data you provide will be very important for furthering treatment for tobacco dependence.

Handling of Collected Information:

All information we collect about you will be kept strictly confidential. This includes questionnaires, information from assessments, and personal identifying information (name, date of birth and OHIP number). At all times, your study data and identifying information will be managed in accordance with Ontario's privacy law (Personal Health Information Protection Act of Ontario). Your data will be kept confidential in two ways. First, information that could be used to identify you is stored separately from all of your study data (e.g., questionnaires, assessments). Second, computer-based security procedures will be applied to all data.

Only authorized research staff at CAMH will have access to information that could identify you or link you with your study data. Unless there is a legal requirement (court order or subpoena), unscrambled information that could identify you will not be given to anyone outside CAMH.

Health Canada has given authorization to conduct this study which involves evaluating a new device. As part of Health Canada's inspection program, their representative may access your records.

This trial will be registered on Clinicaltrials.gov and description of this clinical trial will be available on the website www.clinicaltrials.gov. . This website will not include information that can identify you. At most, the website will include a summary of the results. Anyone can search this website at any time.

Continuing Review

As part of continuing review of the research, your study records may be assessed on behalf of the CAMH Research Ethics Board and by Health Canada Investigational Testing Authorization Programme. A person from the research ethics team may contact you (if your contact information is available) to ask you questions about the research study and your consent to participate. The person assessing your file or contacting you must maintain your confidentiality to the extent permitted by law.
As part of the Research Services Quality Assurance Program, this study may be monitored and/or audited by a member of the Quality Assurance Team. Your research records and CAMH records may be reviewed during which confidentiality will be maintained as per CAMH policies and extent permitted by law.

Payment, Conditions, and Confidentiality:

If you complete all components of the study you will receive total compensation of $670. In addition, you will receive transit tokens to cover the round-trip cost of each visit to the clinic.

You can drop out of the study at any time and receive payment for the parts of the study that you have completed (as outlined above). All information you provide will remain strictly confidential to the extent allowed by law. Your name will not appear on any of the test materials (e.g., questionnaires) or in any of the data from the computer tasks. You will be assigned a subject ID, which will be used to code all of your data. Names and identifying information will be stored in locked cabinets and scrambled electronically. Similarly, any reports of the study findings will be made so that you and all study participants remain anonymous.

Medical Record Number

As part of your participation in this study, the study group facilitator will document key elements of each treatment session, which will be reviewed by the study physician (to monitor study progress), and stored in CAMH's electronic database. This information then becomes part of your health record. In order to record this information, you will receive a unique ***Medical Record Number (MRN)***. You can elect not to receive an MRN. However, without the MRN it will not be possible for a doctor or other CAMH personnel to access your research/treatment history. Having access to this information may be important for guiding future decisions about your care. In this respect, the MRN is a way to help ensure your utmost care and safety. Like all other information about you, the MRN and health record will remain confidential to the extent allowed by law.

Contact

For questions about the purpose of the study or study procedures, please contact Dr. Laurie Zawertailo at 416-535-8501-ext. 77422
For questions about your smoking, or varenicline treatment please contact Dr. Peter Selby at 416-535-8501, ext. 36859
If you have any questions about your rights as a participant in this study, please contact Dr. Padraig Darby, Chair, Research Ethics Board, Centre for Addiction and Mental Health, at 416-535-8501 ext. 36876.

Standard Treatment for Smoking Cessation plus
Transcranial Direct Current Stimulation

- The researcher or a member of the researcher's staff has discussed with me the risks of participation in this study.

- I have read all of the information in the Study Information Sheet, and I have had time to think about the information, and all of my questions have been answered to my satisfaction.

- I voluntarily agree to be part of this research study, to follow the study procedures, and to provide necessary information to the investigator or other staff members as requested.

- I am under no pressure to participate in the study, and I understand that I may withdraw from the study at any time. I also understand that my participation in the study may be terminated by the study investigator/group facilitator if necessary.

- By signing this consent form, I am not giving up my legal rights or releasing the investigators or sponsors from their legal and professional obligations.

- I have received a copy of the Information Sheet and will receive a copy of this signed consent form.

____________________________	__________________	_____________
Print Participant's Name	Participant's OHIP #	Date

____
Participant's Signature

______________________________ _____________
Signature of Individual Obtaining Consent Date

______________________________ _____________
Signature of Investigator Date
(If investigator did not obtain the consent)

Please indicate whether you consent to receiving a CAMH Medical Record Number**:**

I do ☐ OR do NOT ☐ agree to being assigned a CAMH Medical Record Number

Research at CAMH is ongoing and it is often helpful to investigators to contact individuals who have participated in previous studies, who have expressed interest in participating in future research.

Please indicate your interest in being contacted for future studies:
I do ☐ OR do NOT ☐ wish to be contacted for future studies at CAMH.

Payment Details:

The schedule of payment is as follows:

Assessment	$20
Baseline fMRI #1	$70-90
Baseline fMRI #2	$70-90
tDCS Session 1	$20
tDCS Session 2	$20
tDCS Session 3	$20
tDCS Session 4	$20
tDCS Session 5	$20
tDCS Session 6	$20
tDCS Session 7	$20
tDCS Session 8	$20
tDCS Session 9	$20
tDCS Session 10	$20
Booster Session 1	$20
Booster Session 2	$20
Booster Session 3	$20
Booster Session 4	$20
Booster Session 5	$20
End-of-treatment fMRI	$70-90
6-month Follow-up	$50
**Total**	**$580-640**

Health Canada Endorsed Important Safety Information on CHAMPIX (varenicline tartrate)

May 31, 2010

Subject: Important safety information regarding CHAMPIX® (varenicline tartrate)

Pfizer Canada, in collaboration with Health Canada, would like to inform you of important changes to the Product Monograph for CHAMPIX®, including changes to the Consumer Information section.

These changes include:

- The addition of a BOXED WARNING highlighting important recommendations for healthcare professionals regarding information related to neuropsychiatric adverse events;
- A warning regarding rare reports of hypersensitivity reactions, such as angioedema and serious skin reactions, including Stevens-Johnson syndrome and erythema multiforme;
- The addition of an "Information for Patients" section under *WARNINGS AND PRECAUTIONS* providing prescribers with advice regarding key information to be shared with their patients prior to and during treatment; and
- Two dosing options are now approved for CHAMPIX®. Following one week titration, the dose may be increased to a maximum of 1.0 mg twice daily or remain at 0.5 mg twice daily.

CHAMPIX® (varenicline tartrate) is indicated for smoking-cessation treatment in adults in conjunction with smoking-cessation counselling. When prescribing CHAMPIX®, healthcare professionals should discuss with patients its benefits and risks.

There have been continuing Canadian and International post-marketing reports of serious neuropsychiatric symptoms, such as depressed mood, agitation, aggression, hostility, changes in behaviour, suicide related events and worsening of pre-existing psychiatric disorder in patients treated with CHAMPIX®. **These events have occurred in patients with and without pre-existing psychiatric disorder.** Some reported cases may have been complicated by the symptoms of nicotine withdrawal in patients who stopped smoking. Alcohol intake may also increase the risk of patients experiencing psychiatric adverse events during treatment with CHAMPIX®. Patients should stop treatment with CHAMPIX® and contact their healthcare provider immediately if they have, or if their families or caregivers observe, neuropsychiatric symptoms or behaviours that are not typical for the patient.

There have been post-marketing reports of somnolence, dizziness, loss of consciousness, seizures or difficulty concentrating, therefore, patients should be advised not to engage in potentially hazardous activities, such as driving a car or operating dangerous machinery until they know how they may be affected by CHAMPIX®.

There have also been post-marketing reports of patients experiencing hypersensitivity reactions, such as rare life-threatening angioedema events requiring urgent medical attention and rare severe cutaneous reactions, including Stevens-Johnson syndrome and erythema multiforme. Patients should immediately stop treatment with CHAMPIX® and seek emergency medical care if they experience any signs or symptoms of severe skin/hypersensitivity reactions.

There are now two dosing options approved for CHAMPIX®. Following one week titration, the dose may be increased to a maximum of 1.0mg twice daily or remain at 0.5mg twice daily. The dose should be chosen, based on physician judgement of patient tolerance, perceived effectiveness of the treatment, and patient preference. The dosing regimen may be switched temporarily or permanently between these two options, if needed, subsequently.
