# Supplementary material for: Active versus sham transcranial direct current stimulation (tDCS) as an adjunct to varenicline treatment for smoking cessation: Study protocol for a double-blind single dummy randomized controlled trial
Source: PLoS One. 2022 Dec 8;17(12):e0277408. doi: 10.1371/journal.pone.0277408 (PMC9731486; doi:10.1371/journal.pone.0277408)
Supplement: S3 Appendix — Details of biological specimen collection and the tests used for cotinine and drugs abuse. (PDF) [file pone.0277408.s003.pdf]

Appendix S3. Biological Specimens

Urine will be collected periodically through the study to confirm smoking abstinence by using a semi-qualitative cotinine dipstick purchased from Rapid Response Single Drug Test strip (300ng/ml cut off for urinary cotinine). Urine drug screens will also be conducted prior to each MRI scan to confirm absence of psychoactive drugs. This will be done by using the Rapid Response Multi-Drug Integrated Split Specimen Cup, which screens for amphetamines, barbiturates, buprenorphine, benzodiazepines, cocaine, ecstasy, methamphetamine, morphine, methadone, oxycodone, propoxyphene and marijuana.
