# Supplementary material for: Active versus sham transcranial direct current stimulation (tDCS) as an adjunct to varenicline treatment for smoking cessation: Study protocol for a double-blind single dummy randomized controlled trial
Source: PLoS One. 2022 Dec 8;17(12):e0277408. doi: 10.1371/journal.pone.0277408 (PMC9731486; doi:10.1371/journal.pone.0277408)
Supplement: S4 Appendix — Participants will complete the adverse event list at each study appointment. The list consists of the most common side effects of varenicline and tDCS respectively. Participants can indicate the severity by mild, moderate or severe. (PDF) [file pone.0277408.s004.pdf]

Appendix S4. Adverse Events of tDCS and Varenicline

ADVERSE EVENTS FORM

Participant Initials _____ Participant Number __________ Date: ___________
Session #: __________ Form completed by: ___________________________

a. Headache ☐ No ☐ Yes (MILD, MODERATE, SEVERE)
b. Burning sensation on the scalp ☐ No ☐ Yes (MILD, MODERATE, SEVERE)
c. Dizziness ☐ No ☐ Yes (MILD, MODERATE, SEVERE)
d. Itchiness on the scalp ☐ No ☐ Yes (MILD, MODERATE, SEVERE)
e. Rash ☐ No ☐ Yes (MILD, MODERATE, SEVERE)
f. Nausea ☐ No ☐ Yes (MILD, MODERATE, SEVERE)
g. Fatigue ☐ No ☐ Yes (MILD, MODERATE, SEVERE)
h. Trouble sleeping ☐ No ☐ Yes (MILD, MODERATE, SEVERE)
i. Dry mouth ☐ No ☐ Yes (MILD, MODERATE, SEVERE)
j. Vivid dreams ☐ No ☐ Yes (MILD, MODERATE, SEVERE)
k. Other side effect(s) ☐ No ☐ Yes (MILD, MODERATE, SEVERE)

*If yes, please describe the other side effect(s) that you have experienced:

____
____
____

HR: ______________ BP: _____________

Temperature: ______________

Researcher Examination:

Visual Inspection
Signs of redness?
