# Supplementary material for: Active versus sham transcranial direct current stimulation (tDCS) as an adjunct to varenicline treatment for smoking cessation: Study protocol for a double-blind single dummy randomized controlled trial
Source: PLoS One. 2022 Dec 8;17(12):e0277408. doi: 10.1371/journal.pone.0277408 (PMC9731486; doi:10.1371/journal.pone.0277408)
Supplement: S1 File — (DOCX) [file pone.0277408.s005.docx]

**STUDY PROTOCOL**

**Using non-invasive brain stimulation (tDCS) to improve the effectiveness of varenicline for treating tobacco dependence: a randomized controlled trial**

Principal Investigator: Laurie Zawertailo, PhD

Qualified Investigator: Peter Selby, MBBS

Co-investigators: Tarek Rajji, MD PhD

Moshe Eizenman, PhD

**Funding Organization:** Global Research Awards for Nicotine Dependence (GRAND) Pfizer, Inc.

**Site:**

| **Centre for Addiction and Mental Health** |  |
| --- | --- |
| 175 College Street, Toronto, ON M5T 1P7  416-535-8501 |  |

This signature below constitutes the approval of this protocol and provides the necessary assurances that this research study will be conducted according to all stipulations of the protocol, including all statements regarding confidentiality, and according to local legal and regulatory requirements and ICH GCP guidelines.

**Authorized by:**

| **_________________________________** | **_________________________________** |
| --- | --- |
| Laurie Zawertailo Ph.D.  Principal Investigator  CAMH Nicotine Dependence Clinic  175 College St. Toronto 416-535-8501 Ext 77422 | Peter Selby MBBS, CCFP, FCFP, MHSc, Dip ABAM, Qualified Investigator  CAMH Nicotine Dependence Clinic  174 College St Toronto 416-535-8501 Ext 77432 |
|  |  |

**ABSTRACT**

**Rationale**: While varenicline on its own is the most effective medication for smoking cessation, long-term abstinence is still relatively poor. The addition of tDCS as an adjunct to pharmacotherapy is a novel approach but one that is grounded in a growing evidence-base.

**Research Question/Objective**: The primary objective of this research is to provide preliminary evidence of the effectiveness of tDCS as an adjunct treatment to pharmacotherapy for smoking cessation. Does the addition of active tDCS to the left DLPFC improve the effectiveness of varenicline in the treatment of tobacco dependence? Does it make individuals more resistant to relapse in the long-term?

**Study Design**: Double-blind, sham-controlled, randomized clinical trial

**Setting**: A free Nicotine Dependence Clinic in downtown Toronto, Canada

**Participants**: Fifty daily dependent adult smokers seeking treatment to quit smoking

**Intervention and Procedures**: Twelve weeks of varenicline treatment plus tDCS (active: 20 minutes at 2 mA OR sham: 30 seconds at 2 mA, 19.5 minutes at 0 mA), daily (M-F) for the first 2 weeks and then every 2 weeks for the next 10 weeks. 2 FMRI scans at baseline and 1 scan at end-of-treatment. Eye-tracking viewing tests will be conducted at baseline, week 4, week 8,end-of-treatment and at 6 months follow up. Follow-ups at 6-months. A monetary delayed discounting task will be performed at baseline, end of treatment and at 6 months follow up.

**Measurements**: Expired CO, self-reported smoking, and subjective craving and withdrawal at each study visit, weekly self-reported smoking status (7day PPA) from end of treatment to 6 months. For fMRI: BOLD activation, perfusion, structural T1 mapping, DTI and functional connectivity. For Eye-tracking: attentional bias to substance-related cues, attentional bias to negative emotional cues and attentional bias to high-arousal/high-risk cues.

**Potential Impact**: For every two smokers who quit, one life is saved from a tobacco-related mortality. Therefore, even a small improvement in smoking cessation treatment effectiveness and long-term abstinence would decrease the prevalence of tobacco-related death and disease and save the health care system billions of dollars.

**BACKGROUND and RATIONALE**

Despite important strides in the decreasing the prevalence of smoking in North America, one in five adults still smokes tobacco. Current first line medications (nicotine replacement therapy [NRT], bupropion, varenicline) are only effective for some. As such, there has been much interest in optimizing the efficacy of existing treatments. Based on preliminary evidence from a pilot study in our lab using transcranial direct current stimulation (tDCS) as an adjunct to standard nicotine patch treatment, we now hope to demonstrate both the safety and superior efficacy of adding a tDCS treatment regimen to standard varenicline treatment in a randomized sham-controlled double-blind study. The present proposal aims to pilot this protocol in 50 participants as proof-of-concept, and to explore the potential neurobiological and physiological correlates of any treatment effect using magnetic resonance imaging (MRI) and eye-tracking, respectively.

Varenicline is the most effective pharmacotherapy available for the treatment of tobacco dependence (Cahill et al, 2013). However, like all treatments for smoking cessation, successful long-term abstinence is hampered by high rates of relapse (CDC, 2008). Relapse is often reported to be due to succumbing to cravings usually elicited by environmental cues (Janes et al, 2010). This vulnerability to relapse is hypothesized to be due to long-lasting neuroadaptations resulting from chronic exposure to nicotine (Robinson and Berridge, 2001). Specifically, the dorsolateral prefrontal cortex (DLPFC) is thought to exert control over cravings and reward related to smoking (Goldstein and Volkow, 2011). Drug-induced neuroplasticity is a hallmark of addiction (Koob and Volkow, 2010). Smoking cessation may not involve the unlearning of the behavior so much as new learning that can serve to override the old, especially under circumstances of strong craving. Deficits in plasticity induced by nicotine withdrawal (Grundey et al, 2012) could be the mechanism which underlies the long-lasting vulnerability to relapse that plagues many ex-smokers as it impairs the ability of the brain to learn how to decouple pairings between environmental cues and craving (Fraser and Rosen, 2012).

Transcranial direct current stimulation (tDCS) is a form of non-invasive brain stimulation that involves brief (e.g., 20-min) application of weak electric current (e.g., 2 mA) to the scalp. It increases excitability of neurons at the anode with 20-30% of the current going through the brain from anode to cathode so that both cortical and subcortical structures are stimulated. The procedure is very safe, convenient, and fast-acting with well-established parameters and a credible placebo manipulation (‘sham’ tDCS: 30-s stimulation) (Poreisz et al, 2007). It has the ability to modulate plasticity in specific brain areas (Nitsche and Paulus, 2001) and has established effectiveness in human laboratory models of addictive motivation (Boggio et al, 2008; 2009) and has been shown to decrease craving for cigarettes when the anode is placed over the left dorsolateral prefrontal cortex (dlPFC) (Fregni et al, 2008). However, studies to date have only examined the effects of tDCS on laboratory measures of motivation and smoking behavior rather than smoking cessation and relapse prevention in treatment-seeking smokers. The proposed research will begin to address these fundamental gaps.

*Functional activation: cue reactivity:* One of the most prominent models of addiction is incentive sensitization (Robinson and Berridge, 2001), where the brain mesocorticolimbic reward system is sensitized, and drug cues acquire motivational salience and become conditioned reinforces themselves. In the case of smokers, smoking-related environmental stimuli can elicit smoking urges and cravings, which in turn is associated with compulsive smoking and relapse. Most neuroimaging studies examining neural reactivity to smoking-related (compared to neutral) cues have shown that smokers exhibit increased activation in regions involved in reward, craving, emotions, and memory, as well as regions associated with visuospatial attention (Due, 2002), suggesting increased attention to stimuli of heightened attentional salience. One study found that hyper-activation in some of these regions and decreased connectivity with cognitive control networks predicted “slips” in abstinence (Janes et al, 2010), and behavioural and non-nicotinic pharmacological smoking interventions can normalize these brain responses relative to non-smoking controls (Culbertson et al, 2011; Franklin et al, 2011; Westbrook et al, 2013). However, only a small number of studies have examined the effect of varenicline treatment on smoking cue reactivity. Although these studies suggest that varenicline modulates relevant networks, none of these studies used a full 12-week treatment protocol one study only required 3 days of abstinence prior to the second scan (Loughead et al, 2013), and another study required subjects to be non-abstinent even after 3 weeks of medication (Franklin et al, 2011). Our study will address this gap in the literature by conducting fMRI scans at baseline and after 12-weeks of varenicline treatment in both the active and sham treated tDCS groups. The sham tDCS group will allow us to examine the effects 12-weeks of varenicline treatment on cue reactivity in both quitters and non-quitters. Furthermore, we can compare the treatment effects of active versus sham tDCS within-subjects by comparing the pre- and post-treatment scans. Active tDCS should reduce cue-reactivity by increasing plasticity of the left DLPFC.

*Functional activation: reward anticipation:* Most neurological models of addiction stress the importance of the mesocorticolimbic pathways in processing the reinforcing effects of drugs (Koob & Volkow, 2009). Drugs of addiction such as nicotine alter the sensitivity of the brain regions involved in motivation and reward (Robinson & Berridge, 2001). Many studies have shown that addicted individuals show blunted activations in response to non-drug rewards in these brain regions (Balodis & Potenza, 2015; Sweitzer et al., 2015). As a result, addicted individuals with impaired reward processing pathways may contribute to drug-seeking behaviors. Specific brain areas that could be responsible for these changes include: the ventral striatum (Peters et al., 2011), the nucleus accumbens (NAc) (Buhler et al, 2010, Sweitzers et al., 2015) and the anterior cingulate cortex (ACC) (Fedota et al., 2015). Specifically, previous studies have shown that individuals with lower ventral striatal activity during the reward anticipation task were more likely to smoke/relapse (Sweitzer et al., 2015). Another area of interest for nicotine addiction is the medial prefrontal cortex (MPFC), which contributes to goal-directed behaviours in response to reward expectation (Goldstein & Volkow, 2002). Lastly, varenicline has been previously implicated in the reduction of reward signaling and decreasing alcohol intake in humans and rodents (Franklin et al., 2011; Hendrickson et al., 2010). In a recent study, varenicline downregulated gain magnitude processing in the anterior cingulate cortex (ACC), an area associated with attentional control (Fedota et al, 2015). Specifically, varenicline decreased the impact of reward magnitude on ACC activity whereas nicotine increased ACC processing when anticipating a reward (Fedota et al., 2015). Thus, we will be examining differences in the above brain regions at baseline and comparing them with end of treatment scans. The sham tDCS group will allow us to examine the effects 12-weeks of varenicline treatment on reward anticipation in both quitters and non-quitters. Meanwhile, the active tDCS group will allow us to examine the effects of tDCS on reward anticipation compared to non-active tDCS.

**Study Objectives**

The primary objective of this research is to provide preliminary evidence of the effectiveness of tDCS as an adjunct treatment to pharmacotherapy for smoking cessation. The secondary objective is to examine the neurobiological and physiological adaptations that may occur as a result of tDCS treatment. **Research Questions**:

1. Does the addition of active tDCS to the left DLPFC improve the effectiveness of varenicline in the treatment of tobacco dependence? Does it make individuals more resistant to relapse in the long-term?
2. How and what extent does varenicline and tDCS treatment effect brain activity and structures using fMRI?
3. What is the impact of varenicline and tDCS on attentional bias using a validated eye-tracking paradigm?
4. What is the impact of varenicline and tDCS on impulsivity as measured by a validated monetary delayed discounting task?

**Hypotheses**:

1. The combination of varenicline treatment plus tDCS to the left DLPFC will improve smoking cessation outcomes and relapse rates in treatment seeking smokers.
2. Active tDCS will reduce fMRI BOLD response to smoking cues compared to baseline within-subjects and compared to sham tDCS between subjects. Those who quit smoking in the last 4 weeks of treatment will show a “normalization” of brain reactivity to smoking cues and reward anticipation relative to those who were unable to remain abstinent over the short term due to tDCS extinguishing the salient properties of smoking-related cues and reward anticipation.
3. Compared to baseline, varenicline treatment will reduce within subject attentional bias, as measured by parameters of visual scanning behavior (VSB), to smoking and negative emotional cues.
4. Active tDCS will reduce attentional bias to smoking and negative emotional cues compared to sham tDCS. Those who quit smoking in the last 4 weeks of treatment will show “normalized” attentional bias patterns to smoking cues and reward anticipation relative to those who were unable to remain abstinent over the short term. Subjective measures of cravings will be correlated with within-subject changes in attentional bias to smoking cues and reward anticipation.
5. Individuals with larger changes in attentional bias to smoking-related cues and/or negative emotional cues, and lower measures of attentional bias to high-arousal/high-risk cues will have higher rates of abstinence (less relapse). Attentional bias will be measured by parameters of visual scanning behaviour and through the analysis of visual scanning patterns (sequences of fixations).
6. The probability of remaining abstinent over the long term will be inversely correlated with VSB measures of novelty seeking behavior.
7. Active tDCS will reduce impulsivity compared to sham tDCS as measured by higher delayed discounting scores.

**METHODS**

**Design**

This will be a double-blind sham-controlled two-arm randomized clinical trial. Fifty participants will be randomized (1:1) to each arm. Arm 1 will be active tDCS plus varenicline (n=25) and Arm 2 will be sham tDCS plus varenicline (n=25). Treatment duration will be 12 weeks. Subjects will be required to attend an in person assessment visit. They will then be randomized to a condition. Neuroimaging scans (fMRI) and eye-tracking tests will be conducted in all participants at baseline. Participants will undergo two scans at baseline on different days within the same week: one in a satiated state and one in a non-satiated state. The same tasks will be performed at each scan. s. Following the baseline fMRI scans and eye-tracking tests, subjects will start taking the varenicline simultaneous with starting the tDCS regimen whereby they will come to the clinic every day (Monday to Friday) for the first 2 weeks to undergo the stimulation (active: 20 minutes at 2mA, sham: 30 seconds at 2mA and then nothing). Their target quit date will be at the end of this 2 week period. They will return to the clinic every two weeks for the next 10 weeks for a booster tDCS session and to complete questionnaires. Eye tracking tests will be conducted at the end of the fourth and eighth weeks. At the end of the 12-week treatment period they will undergo a third fMRI scan and eye-tracking tests. For the next 14 weeks they will be contacted weekly by e-mail or text message to report on their smoking status as measured by the 7 day point prevalence abstinence question. “Have you had a cigarette, even a puff, in the last 7 days?” At the 6-month time point subjects will be asked to return to the clinic for a brief visit to confirm smoking abstinence using expired CO and urinary cotinine and to complete questionnaires.

**Participants**

Study participants will be 50 treatment seeking daily dependent smokers willing to make a quit attempt in the next 30 days. They will be recruited from our Nicotine Dependence Clinic and from the Greater Toronto Area through paid advertising in local newspapers and through the CAMH Registry and the CAMH website. Participants will have no concurrent Axis I psychiatric disorder (or history of substance use disorder), based on SCID, and be free of psychoactive drugs and medications, based on self-report and urinalysis. To ensure against subtle neurological deficits associated with sub-clinical alcohol misuse, men will be required to drink no more than 12 standard drinks per week and women will be required to drink no more than 9 drinks per week. These values conform to safe drinking limits for non-problem drinking (Sobell and Sobell, 1992). According to standard protocols for tDCS clinical trials, participants will also be excluded if they: a) have had recent clinically significant head trauma; b) have a history of seizures and/or epilepsy; c) are pregnant or planning to become pregnant; d) have pacemakers or implanted electrical devices such as cochlear implants; e) have metal embedded in the skull; or f) have skin lesions, open wounds, bruising, or similar injuries at either of the stimulation sites.

*Eligibility Criteria*

- Male or female
- Ages 19-65
- Daily smoker > 8 CPD
- Seeking treatment to quit smoking
- No current/recent DSM-IV Axis I diagnosis (apart from nicotine dependence)
- No current use of psychoactive drugs or medications
- No current use of Nicotine Replacement Therapy, e-cigarettes, or other medications for smoking cessation
- No history of seizures/epilepsy
- No metal embedded in the skull and/or implanted electrical devices
- No head injury (no concussion – diagnosed by a physician; OR no blow to the head with loss of consciousness for more than 1 hour)
- No known contraindications to fMRI
- No known contraindications to Varenicline
- Able to attend daily appointments for tDCS for the first 2 weeks and then once every 2 weeks for the next 10 weeks.
- Willing to undergo three fMRI scans – two at baseline and another at the end of treatment.

**Procedures**

Following the initial assessment appointment to obtain consent and confirm eligibility, the first tDCS treatment session will be booked. A breathalyser will screen for alcohol and expired CO at each tDCS session. The tDCS stimulation will take place in a quiet room, free of distractions. Stimulation will continue uninterrupted for 20 minutes with the experimenter in the room. While being stimulated, subjects will be required to read self-help materials related to smoking cessation, relapse prevention, and mood management. The readings for each day of stimulation will be standardized for all subjects.

To increase the opportunity to detect an acute beneficial effect of tDCS, participants will be asked to refrain from smoking for 2 hours prior to the start of each treatment session. Relative CO readings from the first day (ad lib smoking) compared to subsequent days will provide a coarse means of verifying compliance with the 2-hour pre-session smoke-free requirement.

Participants will be randomly assigned (1:1) to one of 2 treatment arms (double-blind). Participants will be matched as closely as possible on age, gender and cigarettes per day.

There will be 10 consecutive daily sessions of tDCS with the anode over the left DLPFC and cathode over the right DLPFC (active or sham) (Monday-Friday for 2 weeks; 2 mA x 20 min). Participants assigned to sham will have electrodes placed identically to active but receive only 30 seconds active stimulation. Participants will also be videotaped during each tDCS session to ensure consistency of tDCS application between research personnel and for documenting participant reactions during the session. This will also be used to document any side effects/complications participants may experience during the session. Each video tape session, as well as all data collected in the trial, will be stored on a secure server and will be retained for 10 years after the participant completes the study, as suggested by CAMH’s policy on Storage of Records. After each tDCS session, the video footage will be transferred from the camera to the secure server.

**tDCS**

A battery operated tDCS device (SmartStim Model 1000; Nuraleve, Inc., Sudbury ON) with a maximum 4 mA capacity will be used to administer stimulation. tDCS will be applied by two cutaneous anodal and cathodal electrodes – 35cm^2^ and 100cm^2^, respectively. Each electrode will consist of conductive carbon enclosed in a saline-soaked sponge connected to lead wires attached to the tDCS device. An elasticized headband will hold the electrodes in place. The electrode configuration will be left anodal tDCS following the montage successfully demonstrated in a number of tDCS addiction studies (Boggio et al. 2009; Fregni et al. 2008a). The anode and cathode will be applied to the F3 and F4 regions, respectively, as defined by the standard International EEG 10-20 system (Jasper 1958). Stimulation will consist of 2 mA direct current delivered continuously for 20 min. This conforms to parameters of a recent 5-session study on cigarette craving (Boggio et al., 2009) and numerous other studies with clinical (depressed, substance dependent) and healthy participants (see Feil and Zangen, 2011 for review). Active treatment will consist of a current ramp up over 30 seconds to minimize discomfort, followed by 2mA stimulation over 19 minutes and subsequent ramp down over the final 30 seconds. Sham treatment will include a 30 second ramp up and 30 second ramp down at the beginning and end of the treatment session since tingling is commonly felt at the onset and termination.

**Pharmacological treatment**

Varenicline as per standard prescribing for dose escalation (i.e. Days 1 to 3: 0.5 mg once daily; Days 4 to 7: 0.5 mg twice daily; Days 8 to end of treatment: 1 mg twice daily) will be given concurrently over the same 2 weeks as the tDCS protocol and continue for an additional 10 weeks as per standard treatment duration recommended on the product monograph for a total of 12 weeks of varenicline treatment. Dose adjustments due to adverse events will be allowed (i.e. decrease to 0.5 mg twice daily).

The target quit date for smoking cessation will be on the last day of the two weeks of daily tDCS which corresponds to the recommended quit date after starting varenicline treatment. Subjects will return to the clinic every two weeks for a ‘booster session’ of tDCS. Their mood and smoking behaviour will be assessed and documented including any withdrawal or craving symptoms.

**Management of Adverse Events (AE) and Serious Adverse Events (SAE)**

Side Effects in a study examining the side effects of 567 sessions of tDCS (Poreisz et al. 2007) with 77 healthy controls and 25 patients, tDCS was associated with a mild tingling sensation (70.6%) as well as moderate fatigue (35.3%) and a light itching sensation under the stimulation electrodes (30.4%). After tDCS, a headache (11.8%), nausea (2.9%) and insomnia (0.98%). 21.6% of volunteers experienced a slight burning (1.59±0.91) and 15.7% experienced a mild pain sensation (1.41±0.71) under the electrodes during the stimulation. 17.7% of the volunteers experienced tDCS as mildly unpleasant. The most common side effects of varenicline are nausea, abnormal dreams, constipation, flatulence and vomiting in 30, 13, 8, 6 and 5% of users, respectively. They are reversible and usually not severe. It is not known whether combining the two treatments will increase either the incidence or the severity of adverse effects, especially those that are common between the two treatments such as nausea and headache.

If a participant experiences an adverse event that requires hospitalization or results in lasting harm or death (SAE) and can reasonably be attributed to tDCS stimulation, the trial will be immediately suspended. Adverse events that can be expected based on the known profile of tDCS side effects will be managed by the Qualified Investigator (Dr. Peter Selby). Depending on the severity of the unexpected adverse event, the Qualified Investigator will decide if the trial should be suspended. All SAEs, AEs and UAEs will be reported to the CAMH Research Ethics Board. The emergency contact for reporting SAEs is the Qualified Investigator (Dr. Peter Selby).

**Neuroimaging**

The fMRI neuroimaging component to this study will attempt to answer several research questions and address some of the gaps in the tDCS literature. Specifically, how does tDCS and/or varenicline alter functional connectivity between PFC and amygdala or insula? Does the magnitude of change predict successful cessation and long-term abstinence? Does active tDCS change the BOLD response to smoking cues and/or emotional cues? What is directionality of this effect?

Prior to MRI scans, participants will be required to complete a training session at least 24 hours prior to their first scan using the mock MRI scanner, a device which stimulates the scanner conditions including the screen-response apparatus. MRI tasks will be explained to participants outside the scanner and they will then be able to complete mock versions of the cue-reactivity and reward anticipation tasks. Participants who cannot appropriately complete the tasks or who demonstrate excessive motion or discomfort in the scanner due to the confined environment will be excluded from participation in the study.

Magnetic resonance imaging (MRI) will take place at the CAMH Research Imaging Centre (GE Discovery MR750 3T, General Electric Medical Systems, Milwaukee, WI) on 3 occasions: 1) at baseline following 12 hours of smoking abstinence (abstinence scan) 2) at baseline within 1 hour of smoking a cigarette (satiated scan) and 3) at end of the 12 week varenicline treatment. Baseline scans will occur within 1 week of each other and the order of satiated and abstinent scans will be counterbalanced to control for possible order effects. During each session, we will measure: 1) functional activation (blood-oxygen-level-dependent (BOLD) response) while subjects perform cue reactivity and reward anticipation task; resting state functional connectivity (rsFC) 3) quantitative T1 mapping (qMRI) and/or 4) Diffusion Tensor Imaging (DTI).

*Measures at rest*

The following measures (rsFC, DTI, qT1) will be collected with the subject at rest, i.e., no task performance will be required. qT1 images will be acquired across the whole brain. Diffusion Tensor Imaging (DTI)

DTI is a widely used MRI technique for measuring water diffusion within specific areas of the brain by quantifying the intensity of each image element, which in turn, reflects the rate of water diffusion. DTI is a useful measure for identifying white matter microstructures and has been shown to significantly alter brain tractography in adult smokers in areas such as the corpus callosum (Paul et al., 2008, Hudkins et al., 2012, Lin et al., 2012). DTI will be used to test for significantly tractography and/or white matter differences that may exist between active tDCS vs sham.

Resting state functional connectivity (rsFC)

rsFC is a task-independent measure of brain activity that is based on correlations between low frequency fluctuations of BOLD signal between brain regions in the absence of an explicit task (Biswal et al., 1995). Smoking has been shown to affect rsFC in the ventral and dorsal striatum, the insula and limbic systems (Ding et al., 2013, Janes et al., 2012, Sweitzer et al., 2016). rsFC data will be used to evaluate if there are differences in rsFC between active tDCS and sham and also if these rsFC networks are correlated with quit at end of treatment.

*Brain structure:* Structural MRI studies have identified cortical thickness, white matter, and volumetric abnormalities in smokers in cerebellum, striatum, cingulate cortex, prefrontal cortex, and insula using voxel-based morphometry (VBM) (see (Azizian et al, 2009) for review), and these, in turn, relate to cigarette exposure and craving (Morales et al, 2014). Quantitative T1-weighted MRI measures (qMRI) can shed additional light on the tissue microstructure underlying these findings, and allows for quantification (rather than only visualization) of results. These measures will be used to assess and detect subtle brain tissue alterations in both grey and white matter before and after tDCs treatment and/or smoking cessation. These neuroimaging parameters have never been assessed for changes resulting from abstinence from smoking nor have they been assessed in the context of tDCS treatment.

Each MRI scan will be preceded by an assessment of withdrawal, mood, and craving (Questionnaire of Smoking Urges (QSU; Tiffany & Drobes, 1991); POMS (Profile of Mood States)). Breathalyzer tests and urine samples will be collected at all scans to confirm abstinence from alcohol and other drugs of abuse prior to scans

**Cognitive Tasks**

*Cue reactivity paradigm*

In this task, subjects are presented with a series of smoking-related and neutral photographic cues, adopted from the International Smoking Image Series (Gilbert et al, 1999). Smoking cues include photographs of people smoking and smoking-related objects such as packs of cigarettes and ashtrays. Neutral cues consist of neutral themed pictures selected not to elicit strong emotional responses, such as neutral faces, flowers, or furniture. Visual cues will be presented in a block design, with 4 pictures/block, lasting a total of 20s (5s/picture). There will be 8 blocks of neutral and smoking cues each, presented in a random order, and all pictures presented within each block will be randomized. Each picture block will be interspersed with a rest fixation cross block lasting 18s.

*Monetary Reward Anticipation Task Paradigm*

This task was designed by Garrison et al. (Garrison et al., 2017), which was modified version of the MIDF from Knuston et al. (Knuston et al., 2000). This task was designed based on evidence that drugs of addiction, such as nicotine, alters brain sensitivity in regions specifically involved in motivation and reward (Robinson et al., 2001). Specifically, chronic drug use is associated with responsibility to drug related cues in the mesocorticolimbic dopaminergic reward circuit in the ventral striatum and medial prefrontal cortex (Koob et al., 2010). Thus, impairment in reward processing in addiction could contribute to the drug seeking behaviour and decrease treatment effectiveness. Participants are primed for 6 seconds with possible shapes representing condition cue-indicators, which indicate the possibility of monetary gain, monetary loss, verbal feedback (procedural control), or request no response (control). Immediately following the cue image presentation, a prespecified stimulus will prompt participants to respond as quickly as possible to either: receive a reward, prevent reward loss or receive feedback on their performance. The reaction time required for a registered response is adapted on a trial-by-trial basis such that all participants will receive similar earnings by the end of the task. Participants will practice the task prior to scans to minimize learning effects. The participants will receive a maximum of $20 for each time they do this task.

**Eye Tracking**

The eye tracking component to this study will attempt to answer several research questions. Specifically, does the magnitude of within-subject changes in VSB to smoking cues/and or emotional cues predict successful cessation and long-term abstinence? Does active tDCS change VSB to smoking cues, negative emotional cues or high-arousal/high-risk cues? Are within-subject changes in VSB to smoking/and or emotional cues correlate with the individual’s cravings? Does the individual’s VSB to high-arousal/high-risk cues correlate with long term treatment outcome? Eye movements will be recorded by Visual Attention Scanning Technology (VAST) (EL-MAR Inc. Toronto, Ontario Canada) (Guestrin and Eizenman, 2006, Pinhas et al., 2014, Chau et al., 2016) on five separate occasions (baseline, 4-weeks after start of treatment, 8-weeks after start of treatment,at the end of treatment -12-weeks and 6 months follow up). During each session, we will measure: 1) attentional bias to smoking cues, 2) attentional bias to negative emotional cues, and 3) attentional bias to high-arousal/high-risk cues.

**Free Viewing paradigm**

The eye tracking tests will use the free viewing paradigm for attentional bias assessment that was developed by Eizenman et al., (2003). In this paradigm individuals freely view (i.e., no implied task) multiple visual stimuli that are presented simultaneously and compete for the individual’s attention. Under free viewing conditions, visual scanning behaviour (VSB) is reflective of internal thought processes and closely follow shifts in attention (Kowler, 1995, Posner & Dehaene, 1994). The free viewing paradigm was used to assess attentional bias in studies of patients with eating disorders, mood disorders and Alzheimer disease (Fok, 2012, Pinhas et al, 2014, Chau et al, 2016, Chung, 2015).

*Approximate time for an eye-tracking session*

Setup and subject calibration: 3 min
Attentional bias to substance-related cues: 5 min
Attentional bias to negative emotional cues: 5 min

Attentional bias to high-arousal/high-risk cues: 5 min

Total test time: 18 min

Each test will be preceded by an assessment of withdrawal, mood, and craving (Questionnaire of Smoking Urges (QSU); Tiffany & Drobes, 1991) and an assessment of mood (Profile of Mood States, POMS). During the baseline assessment participants will also complete a questionnaire on sensation seeking behaviour (Sensation Seeking Scale, Zuckerman et al., 1978).

**Eye-Tracking Method**

The visual attention scanning technology (VAST) includes a binocular eye tracking system (Guestrin and Eizenman, 2006) that records eye-gaze positions, a monitor to display visual stimuli, software to process and estimate visual scanning parameters and a monitoring station to control and supervise the progress of the study (Pinhas et al, 2014, Chau et al, 2016). Processing of eye gaze data includes the segmentation of gaze-position data to fixation sequences, the association of fixations with areas-of-interest on the visual stimuli and the calculations of visual scanning parameters (Pinhas et al, 2014, Chau et al, 2016).

During the test, participants sit at a distance of approximately 65 cm from the monitor and are allowed to move their head freely within a relatively large volume (25x25x25 cm^3^). Subjects complete a short (30 sec) eye tracking calibration step in which participants follow a moving target on the screen. Following calibration, participants view a series of 85 slides that are presented on the monitor of the VAST system. Each slide contains 4 images that are arranged in a 2 by 2 configuration. The visual angle subtended by each of the four images (at the 65 cm viewing distance) is approximately 15.5° × 12.2° and the horizontal and vertical separation between any two images is greater than 2.5°. Similar to previous studies (Pinhas et al, 2014, Chau et al, 2016, Chung et al, 2015), 48 slides will be used as test slides (these slides are used to test attentional biases to smoking cues, negative emotional cues and high-arousal/high-risk cues), 32 filler slides will be used to mask the purpose of the experiment, and 5 practice slides will be used at the beginning of the test to familiarize subjects with the procedure. Each slide is presented for 10.5 seconds and the total testing time/subject is approximately 18 minutes.

*Attentional bias to substance-related cues*

Individuals with substance use disorders typically show an attentional bias to substance-related cues. The attention bias is thought to develop as a consequence of a classical conditioning process. One general model of addiction (Franken et al, 2003) suggests that perception of substance-related stimuli lead to increased dopamine level in corticostriatal circuits that draw the subject’s attention towards the perceived substance. The increase in attention promotes craving that further increase the attentional bias. The combination of increased attentional bias and craving increase the probability of substance use and relapse. An Attentional bias towards substance related cues also lead to increase in substance related cognitions (obsessive thoughts about the substance) and to decrease in the ability to execute coping or control strategies that are based on other cues. These two processes work together to increase and maintain an attentional bias towards substance related cues. Most models of addiction (Field et al, 2014) suggest that a) attentional bias for substance-related cues is a characteristic of substance use disorders, b) the bias is associated with the strength of craving at that moment in time and c) strong attentional bias increase the risk of relapse to substance use in those attempting to remain abstinent or to reduce their substance use.

A common paradigm to study attentional bias in substance use disorders is to present subjects with neutral images (desks, pens, etc.) and substance-related images and measure differences between behaviors (reaction time, eye movements) and/or neural activity (EEG, fMRI) when these two classes of stimuli are viewed. We will use the free viewing paradigm and 16 slides to study attentional bias to smoking related cues. Each slide will include an image of smoking related cues (cigarettes, people smoking), an image of a neutral object and two high valence images. The positions (top-left, top-right, bottom-left and bottom-right) of the three different images will be randomly distributed between the slides.

*Attentional bias to negative emotional cues*

According to the affective processing model of negative reinforcement, escape or avoidance of negative affect caused by withdrawal is the principal motive for relapse and renewed smoking (Baker and Fiore, 2004). According to this model as negative affect grows with the length of abstinence it produces biases in attentional processes so that the individual’s attention tends to focus more and more on perceived emotional threats (Baker and Fiore, 2004, Kwak et al., 2007). By monitoring biases in attentional processing to negative or emotionally threatening visual stimuli one can provide an indirect measure of the magnitude of negative affect (the aversive state) in an individual who attempt to withdrawal from smoking. Since the amygdala is integral to the regulation of negative emotions, threatening and sad facial expressions that are often used to elicit amygdala reaction (Haxby et al, 2002) will be used with the free viewing paradigm to study attentional bias to negative emotional cues. We will use 16 slides, where each slide includes an image of a threatening face, a sad face, a happy face and a neutral face. The positions (top-left, top-right, bottom left and bottom right) of the four different expressions will be randomly distributed between the slides. We will use the outcome measures of this test to investigate and compare the treatment effects of varenicline and active tDCS on attentional bias to negative emotions in different sessions. We will also examine the relationship between changes in emotional bias and craving.

*Attention bias to high arousal/high risk cues*

Sensation seeking behavior is common in people with substance use disorders and is correlated with vulnerability to substance addiction (Wingo et al, 2016). Sensation seeking behavior is believed to have a biological basis that is expressed as a need for physiological arousal, novel experience, and a willingness to take social, physical, and financial risks to obtain such arousal (Zuckerman et al, 1979, Bardo et al, 1996). The severity of mal-adaptive patterns of novelty seeking behavior are correlated with substance abuse (Wingo et al, 2016, Stacy et al, 1993) and with vulnerability to the initiation of substance self-administration and compulsive substance use (Belin et al, 2012). Moreover, some studies suggest that sensation seeking behavior can predict treatment outcome and relapse (Ismael et al, 2014, Kahler et al, 2009). We hypothesize that sensation seeking behaviour, as measured by attentional bias to high -arousal/high-risk cues, will be correlated with the probability of remaining abstinent over the long term. To test this hypothesis we will use the free viewing paradigm and 16 slides. Each slide will include an image with high-arousal/high risk scenario, image of a neutral object and two images with positive high valence scenarios. The positions (top-left, top-right, bottom-left and bottom-right) of the three different images will be randomly distributed between the slides. We will study the correlation between attentional bias to high arousal/high risk cues and the standard assessment of sensation seeking behavior (Zuckerman et al., 1978), and also the extent to which tDCS affect novelty seeking behaviour.

**Monetary Delayed Discounting Task**

Individuals can have different discounted values of delayed rewards. In other words, delayed rewards are often interpreted as less valuable compared to an immediate rewards. This phenomenon is called delayed discounting. As such, steep discounters (low value for a delayed reward) are more impulsive than shallow discounters. Numerous previous studies have shown that individuals that smoke, drink or use other drugs of abuse, tend to discount delayed rewards more steeply than non-users (MacKillop et al, 2011; Yi et al., 2010). Delayed discounting is measured by a 27 Item Questionnaire developed by Kirby et al. (1999), a questionnaire that has been validated and used in other studies (Kirby et al., 2004; Myerson et al., 2014). Participants will be completing this survey at baseline, end of treatment and at 6 months follow up.

**Participant compensation**

Participants will be compensated as follow: $20 for baseline assessment (1), $20 per tDCS visit (15 total sessions), $95 per fMRI scans (3 sessions total) and $50 for 6 month follow up visit (1).

**Outcome Measures**

The primary outcome measure will be continuous abstinence from smoking during weeks 9 – 12 as reported at clinic visits during those 4 weeks and confirmed using expired CO. Subjects will be classified as treatment responders if they respond “no” to the question: Have you smoked any cigarettes (even a puff) in the last seven days?

The key secondary outcome measures are:

1. Self-reported 7 day point prevalence abstinence throughout weeks 2 to 12 confirmed by expired CO at each clinic visit. To calculate survival curves we will also collect self-reported continuous abstinence weeks 9 through 26. For weeks 13 to 25, this will be collected via email or text message. For week 26, subjects will attend a brief clinic visit so abstinence can be confirmed with expired CO and urinary cotinine using a semi-quantitative dipstick. We hypothesize that the active tDCS group will have higher rates of abstinence (less relapse) than the sham tDCS group.

2. fMRI: change in Blood Oxygen Level Dependent (BOLD) activation in brain regions known to be associated with drug craving and emotion processing/regulation. MRS: GSH levels measured in a single voxel placed over prefrontal cortex. Fractional anisotropy (FA) values will be extracted from DTI analysis from regions of interest (ROIs) or to form probabilistic tractography for the whole brain. Network maps will be extracted and analyzed from the resting state functional connectivity measure.

3. Eye Tracking tests: changes in attentional bias to smoking-related cues and negative emotional cues. Attentional bias to high-arousal/high-risk cues.

*Self-Report Measures*

Participants will complete the 10-item Questionnaire of Smoking Urges-Brief (QSU-10; Tiffany and Drobes, 1991) and the Minnesota Nicotine Withdrawal Scale (MNSW; Toll et al, 2007) at each treatment session upon arrival and immediately after tDCS. They will complete a Side Effects Checklist (Zawertailo et al., 1995) at the end of each treatment session.

Trait scales to be completed at baseline will include the FTND (Heatherton et al 1991) and Alcohol Use Disorders Identification Test (Saunders et al., 1993), 90-day Alcohol Timeline Followback (TLFB; Sobell and Sobell, 1992); 30-day Tobacco Timeline Followback (adapted from alcohol TLFB); Sensation Seeking Scale (Zuckerman et al., 1978).

**Sample Size and Power Calculation**

If we assume a 40% abstinence rate at end of treatment in the varencline plus sham tDCS group, the abstinence rate in the active tDCS group would need to be double that or 80% in order to see a statistically significant difference with n=25 per arm, p <0.05, and 80% power. As such we do not expect to see a statistically significant difference between the two treatment arms at 25 subjects per arm.

For the treatment part of the study, the main purpose of the study is to establish an Effect Size for tDCS as an adjunct to standard varenicline treatment. This is independent of the number of subjects but allows us to estimate the N that would be required to achieve statistical significance depending on the effect size we observe. The effect size measure we will use will be the partial η2 generated by SPSS and is described in greater detail below.

For the MRI portion of the study, 15 to 25 subjects per group is standard in the neuroimaging field and is more than sufficient to detect significant within- and between-group differences in brain activity.

**Data Analysis**

Continuous data (cravings, mood, cigarettes smoked/day, CO) will be assessed with 2 Gender x 2 (Laterality) x 10 (session) Analyses of Variance (ANOVA). Parallel ANOVAs will assess self-report measures during post-treatment follow-up. Categorical data (e.g., # abstinent participants/session) will be assessed with chi-square, and Kaplan-Meyer survival analysis for repeated measures. Planned comparisons will be performed for the critical experimental conditions (active tDCS) vs. sham.

The critical metric will be Effect Size (partial η2 generated by SPSS), rather than p level. This will reveal the degree of efficacy for the primary outcome variable, Consecutive Days Abstinent (small = .01, medium = .06, large = .14), and allow us to estimate N required to achieve statistical significance for this effect (80% power, α = .05, two-tailed). Pearson correlations will explore the possible relationship between effects of tDCS (session 10 scores, controlling for session 1) and trait factors (e.g., FTND, age, gender, depression).Analysis of MRI data

For the neuroimaging data, brain imaging measures will be compared between groups and across time points, using analysis of variance. MRI measures will also be related to subjective responses (i.e., baseline scores and changes in craving, mood, and withdrawal) using linear regression.

fMRI data will be analyzed by fitting a general linear model (GLM) to the data time-series at every voxel across the brain (and/or within ROIs) and assessing effects using F/t-tests and % signal change calculations. Quantitative T1 maps and DTI analysis will be analyzed at the whole brain level and using ROIs.

After back-sorting by treatment outcome (achieved/maintained abstinence), we expect baseline measures to predict relapse, and active tDCS treatment to result in more efficient functional activation, . than sham tDCS at the second MRI scan.

Analysis of Eye-Tracking Data

For the eye-tracking data, gaze position data are processed by VAST into sequences of fixations and saccades and summary statistics of visual scanning parameters are computed. The VAST parameters include: the number of fixations, the average fixation duration, the average saccadic amplitude and the number of transitions for each image type. These parameters are sensitive measures of attentional bias for different visual cues. The outcome measures of this paradigm will be analyzed using a mixed-model analysis of variance (ANOVA). The within-groups factors are the image types (smoking, neutral, high valence) and session (sessions 1, 2, 3,4 and 5) and the between-groups factor is group (active tDCS and sham tDCS). Pearson’s product–moment correlations will be conducted between parameters of attentional bias and subjective craving scores. Through analysis, we will investigate and compare the treatment effects of varenicline and active tDCS on attentional bias in different sessions. We will also examine the relationship between changes in attentional bias and craving.

By means of a mixed design repeated-measures ANOVA, the VAST parameters will be compared within-subjects at 5 sessions to investigate the effects of varenicline treatment. The parameters will also be compared between the two groups of subjects to examine the treatment effects of active tDCS. We will also compare the VSB parameters of those who quit smoking in the last 4 weeks of treatment and those who were unable to remain abstinent to find out the possible indicators of treatment outcome. Pearson correlation method will be used to explore the relationships between: a) subjective craving and VSB parameters, b) sensation seeking behavior as measured by the sensation seeking scale measures and VSB parameters. Also we will use recurrent neural networks (Chung et al, 2018) to analyze differences between fixation sequences of patients that were treated with tDCS and patients that were not, and between patients that quit smoking and those that were not. Using Cox regression of survival analysis, we will also investigate the correlation between the length of abstinence (time to relapse) and VSB parameters associated with smoking cues, negative emotional cues and high-arousal/high-risk cues.

All data analysis will be conducted using SPSS, SPM or R as appropriate.

**REFERENCES**

Addicott MA, Froeliger B, Kozink R V, Van Wert DM, Westman EC, Rose JE, et al. Nicotine and Non-Nicotine Smoking Factors Differentially Modulate Craving, Withdrawal and Cerebral Blood Flow as Measured with Arterial Spin Labeling. Neuropsychopharmacology. 2014 May 13;

Azizian A, Monterosso J, O’Neill J, London ED. Magnetic resonance imaging studies of cigarette smoking. Handb Exp Pharmacol. 2009 Jan;(192):113–43.

Baker, T.B.,M.E. Piper,D.E. McCarthy,M.R. Majeskie.M.C. Fiore, Addiction motivation reformulated: an affective processing model of negative reinforcement. Psychological review, 2004. 111(1): p. 33.

Balodis, Iris M., and Marc N. Potenza. “Anticipatory Reward Processing in Addicted Populations: A Focus on the Monetary Incentive Delay Task.” *Biological Psychiatry*, vol. 77, no. 5, 2015, pp. 434–444., doi:10.1016/j.biopsych.2014.08.020.

Bardo MT, Donohew R, Harrington NG. Psychobiology of novelty seeking and drug seeking behavior. Behavioural brain research 1996; 77: 23-43.

Belin, D.,V. Deroche-Gamonet, Responses to novelty and vulnerability to cocaine addiction: contribution of a multi-symptomatic animal model. Cold Spring Harbor perspectives in medicine, 2012. 2(11): p. a011940.

Boggio PS, Liguoric P, Sultanic N, Rezendea L, Fecteau S, Fregni F. Cumulative priming effects of cortical stimulation on smoking cue-induced craving. Neuroscience Letters 2009;463:82–86

Boggio PS, Sultani N, Fecteau S, Merabet L, Mecca T, Pascual-Leone A, Basaglia A, Fregni F. Prefrontal cortex modulation using transcranial DC stimulation reduces alcohol craving: a double-blind, sham-controlled study. Drug Alcohol Depend. 2008; 92(1-3):55-60.

Brandon TH, Baker TB The Smoking Consequences Questionnaire: the subjective expected utility of smoking in college students. J Clin Consult Psychol 1991;3:484–491

Buhler M, Vollstadt-Klein S., Kobiella A., Budde H., Reed LJ., Braus DF., Buchel C., Smolka MN. Nicotine Dependence Is Characterized by Disordered Reward Processing in a Network Driving Motivation. *Yearbook of Psychiatry and Applied Mental Health,* *2011*, 106-107.

Burklund LJ, Creswell JD, Irwin MR, Lieberman MD. The common and distinct neural bases of affect labeling and reappraisal in healthy adults. Front Psychol. Frontiers; 2014 Jan 24;5:221.

Cahill K, Stevens S, Perera R, Lancaster T. Pharmacological interventions for smoking cessation: an overview and network meta-analysis. Cochrane Database Syst Rev. 2013:5:CD009329.

Carmody TP, Vieten C, Astin J a. Negative affect, emotional acceptance, and smoking cessation. J Psychoactive Drugs. 2007 Dec;39(4):499–508.

Centers for Disease Control. Cigarette smoking among adults – United States, 2007. MMWR. 2008;57:1221-26.

Chau, S.A.,J. Chung,N. Herrmann,M. Eizenman.K.L. Lanctot, Apathy and attentional biases in Alzheimer’s disease. Journal of Alzheimer's Disease, 2016. 51(3): p. 837-846.

Chung, J.M.K., Visual scanning behaviour in patients with alzheimer's disease 2015.

Chung, J., Eizenman, M., Rakita, U., McIntyre S. R. & Giacobbe P. (2018). Learning Differences between Visual Scanning Patterns can Disambiguate Bipolar and Unipolar Patients. AAAI conference on Artificial Intelligence 2018.

Culbertson CS, Bramen J, Cohen MS, London ED, Olmstead RE, Gan JJ, et al. Effect of bupropion treatment on brain activation induced by cigarette-related cues in smokers. Arch Gen Psychiatry. 2011 May;68(5):505–15.

Curran, S.L.,M.A. Andrykowski.J.L. Studts, Short form of the Profile of Mood States (POMS-SF): Psychometric information. Psychological assessment, 1995. 7(1): p. 80.

Domino EF, Minoshima S, Guthrie S, Ohl L, Ni L, Koeppe RA, et al. Nicotine effects on regional cerebral blood flow in awake, resting tobacco smokers. Synapse. 2000 Dec 1;38(3):313–21.

Due DL. Activation in Mesolimbic and Visuospatial Neural Circuits Elicited by Smoking Cues: Evidence From Functional Magnetic Resonance Imaging. Am J Psychiatry. American Psychiatric Association; 2002 Jun 1;159(6):954–60.

Eizenman, M.,H.Y. Lawrence,L. Grupp,E. Eizenman,M. Ellenbogen,M. Gemar.R.D. Levitan, A naturalistic visual scanning approach to assess selective attention in major depressive disorder. Psychiatry research, 2003. 118(2): p. 117-128

Fedota, J. R., Sutherland, M. T., Salmeron, B. J., Ross, T. J., Hong, L. E., & Stein, E. A.. Reward Anticipation Is Differentially Modulated by Varenicline and Nicotine in Smokers. *Neuropsychopharmacology,2015 40*(8), 2038-2046. doi:10.1038/npp.2015.54

Field M, Marhe R, Franken IH. The clinical relevance of attentional bias in substance use disorders. CNS spectrums 2014; 19: 225-230.

Fok, K.-H. Analysis of Visual Scanning Behaviours for the Objective Assessment of Psychiatric Disorder. 2012.

Franken IH. Drug craving and addiction: integrating psychological and neuropsychopharmacological approaches. Progress in Neuro-Psychopharmacology and Biological Psychiatry 2003; 27: 563-579.

Franklin T, Wang Z, Suh JJ, Hazan R, Cruz J, Li Y, et al. Effects of varenicline on smoking cue–triggered neural and craving responses. Arch Gen Psychiatry. 2011 May;68(5):516–26.

Franklin TR, Shin J, Jagannathan K, Suh JJ, Detre JA, O’Brien CP, et al. Acute baclofen diminishes resting baseline blood flow to limbic structures: a perfusion fMRI study. Drug Alcohol Depend. 2012 Sep 1;125(1-2):60–6.

Fregni F, Liguori P, Fecteau S, Nitsche MA, Pascual-Leone A, Boggio PS. Cortical stimulation of the prefrontal cortex with transcranial direct current stimulation reduces cue-provoked smoking craving: a randomized, sham-controlled study. J Clin Psychiatry. 2008; 69(1):32-40.

Fraser PE, Rosen AC. Transcranial direct current stimulation and behavioral models of smoking addiction. Front Psychiatry 2012;3:doi: 10.3389/fpsyt.2012.00079.

Froeliger B, Modlin L, Wang L, Kozink R V, McClernon FJ. Nicotine withdrawal modulates frontal brain function during an affective Stroop task. Psychopharmacology (Berl). 2012 Apr;220(4):707–18.

Garrison, K. A., Yip, S. W., Balodis, I. M., Carroll, K. M., Potenza, M. N., & Krishnan-Sarin, S. Reward-related frontostriatal activity and smoking behavior among adolescents in treatment for smoking cessation. *Drug and Alcohol Dependence 2017;* *177*, 268-276.

Gilbert DG, Rabinovich NE. International smoking images series (with neutral counterparts), (version 1.2 Carbon-dale) Southern Illinois University: Integrative Neuroscience Laboratory, Department of Psychology; 1999.

Goldstein RZ, Volkow ND. Dysfunction of the prefrontal cortex in addiction: neuroimaging findings and clinical implications. Nat Rev Neurosci. 2011; 12(11):652-69.

Grundey R, Thirugnanasambandam N, Kaminsky K, Drees A, Skwirba AC, Lang N, Paulus W, Nitsche MA. Neuroplasticity in cigarettes smokers is altered under withdrawal and partially restituted by nicotine exposure. J Neurosci 2011;32:4156-62.

Guestrin ED, Eizenman E. General theory of remote gaze estimation using the pupil center and corneal reflections. Biomed Eng IEEE Trans On. 2006;53:1124–33.

Hariri AR, Bookheimer SY, Mazziotta JC. Modulating emotional responses: effects of a neocortical network on the limbic system. Neuroreport. 2000 Jan 17;11(1):43–8.

Haxby J V, Hoffman EA, Gobbini MI. Human neural systems for face recognition and social communication. Biol Psychiatry. 2002 Jan 1;51(1):59–67.

Heatherton TF, Kozlowski LT, Frecker RC, Fagerstrom KO The Fagerstrom test for nicotine dependence: a revision of the Fagerstrom Tolerance Questionnaire. Br J Addict 1991 86: 1119–1127

Ismael, F.,D.A. Baltieri, Role of personality traits in cocaine craving throughout an outpatient psychosocial treatment program. Revista Brasileira de Psiquiatria, 2014. 36(1): p. 24-31.

Janes AC, Pizzagalli DA, Richardt S, deB Frederick B, Chuzi S, Pachas G, et al. Brain reactivity to smoking cues prior to smoking cessation predicts ability to maintain tobacco abstinence. Biol Psychiatry. 2010 Apr 15;67(8):722–9.

Kahler, C.W.,N.S. Spillane,J. Metrik,A.M. Leventhal.P.M. Monti, Sensation seeking as a predictor of treatment compliance and smoking cessation treatment outcomes in heavy social drinkers. Pharmacology Biochemistry and Behavior, 2009. 93(3): p. 285-290.

Kamarck TW, Lichtenstein E. Program adherence and coping strategies as predictors of success in a smoking treatment program. Health Psychol. 1988 Jan;7(6):557–74.

Kirby K & Petry, N. Heroin and cocaine abusers have higher discount rates for delayed rewards than alcoholics or non-drug-using controls. Addiction, 2004. 99 (4): p461-471.

Kirby K., Petry, N., Bickel Heroin addicts have higher discount rates for delayed rewards than non-drug-using controls. J Exp Psychol Gen, 1999. 128(1): p78-87.

Kwak SM, Na DL, Kim G, Kim GS, Lee JH. Use of eye movement to measure smokers' attentional bias to smoking-related cues. Cyberpsychology & Behavior the Impact of the Internet Multimedia & Virtual Reality on Behavior & Society 2007; 10: 299.

Koob GF, Volkow ND. Neurocircuitry of addiction. Neuropsychopharmacol 2010;35(1):217-38.

Lieberman MD, Eisenberger NI, Crockett MJ, Tom SM, Pfeifer JH, Way BM. Putting feelings into words: affect labeling disrupts amygdala activity in response to affective stimuli. Psychol Sci. 2007 May;18(5):421–8.

Lieberman MD, Inagaki TK, Tabibnia G, Crockett MJ. Subjective responses to emotional stimuli during labeling, reappraisal, and distraction. Emotion. American Psychological Association; 2011 Jun 1;11(3):468–80.

Loughead J, Ray R, Wileyto EP, Ruparel K, O’Donnell GP, Senecal N, et al. Brain activity and emotional processing in smokers treated with varenicline. Addict Biol. 2013 Jul;18(4):732–8.

Lyvers M, Carlopio C, Vicole Bothma H, Edwards MS. Mood, mood regulation, and frontal systems functioning in current smokers, long-term abstinent ex-smokers, and never-smokers. J Psychoactive Drugs. Routledge; 2014 Jan 6;46(2):133–9.

MacKillop J, Amlung MT, Few LR, Ray LA, Sweet LH, Munafò MR. Delayed reward discounting and addictive behavior: a meta-analysis. Psychopharmacology. 2011;216:305–321. doi: 10.1007/s00213-011-2229-0

Myerson J., Baumann AA., Green L. Discounting of delayed rewards: (A) Theoretical interpretation of the Kirby Questionnaire. Behav Processes. 2014; 107; 99-105.

Mihov Y, Hurlemann R. Altered amygdala function in nicotine addiction: insights from human neuroimaging studies. Neuropsychologia. 2012 Jul;50(8):1719–2

Morales AM, Ghahremani D, Kohno M, Hellemann GS, London ED. Cigarette exposure, dependence, and craving are related to insula thickness in young adult smokers. Neuropsychopharmacology. 2014 Jul;39(8):1816–22.

Nitsche MA, Paulus W. Sustained excitability elevations induced by transcranial DC motor cortex stimulation in humans. Neurology, 2001 57(10): 1899-1901.

Onur O a, Patin A, Mihov Y, Buecher B, Stoffel-Wagner B, Schlaepfer TE, et al. Overnight deprivation from smoking disrupts amygdala responses to fear. Hum Brain Mapp. 2012 Jun;33(6):1407–16.

Payer DE, Baicy K, Lieberman MD, London ED. Overlapping neural substrates between intentional and incidental down-regulation of negative emotions. Emotion. 2012 Apr;12(2):229–35.

Petcharunpaisan S, Ramalho J, Castillo M. Arterial spin labelling in neuroimaging. World Journal of Radiology. 2010 Oct;2(10):384-98.

Peters, Jan, et al. Lower Ventral Striatal Activation During Reward Anticipation in Adolescent Smokers. *American Journal of Psychiatry* 2011; pp. 540–549.

Pinhas L, Fok K-H, Chen A, Lam E, Schachter R, Eizenman O, et al. Attentional biases to body shape images in adolescents with anorexia nervosa: An exploratory eye-tracking study. Psychiatry Res. 2014;220(1):519–26.

Poreisz C, Boros K, Antal A, Paulus W. Safety aspects of transcranial direct current stimulation concerning healthy subjects and patients. Brain Research Bulletin 2007;72(4-6):208-214.

Robinson TE, Berridge KC. Incentive-sensitization and addiction. Addiction 2001 Jan;96(1):103–14.

Rose JE, Behm FM, Westman EC, Mathew RJ, London ED, Hawk TC, et al. PET studies of the influences of nicotine on neural systems in cigarette smokers. Am J Psychiatry. 2003 Feb;160(2):323–33.

Saunders JB, Aasland OG, Babor TF, de la Fuente JR, Grant M. Development of the Alcohol Use Disorders Identification Test (AUDIT): WHO Collaborative Project on Early Detection of Persons with Harmful Alcohol Consumption--II. Addiction 1993 88:791-804.

Sobell LC, Sobell MB Timeline Followback: a technique for assessing self-reported alcohol consumption. In: Litten RZ, Allen J (eds). Measuring Alcohol Consumption: Psychosocial and Biological Methods. Humana: Totowa, NJ. 1992 pp 41–72.

Stacy AW, Newcomb MD, Bentler PM. Cognitive Motivations and Sensation Seeking as Long-Term Predictors of Drinking Problems. Journal of Social & Clinical Psychology 1993; 12: 1-24.

Sutherland MT, Carroll AJ, Salmeron BJ, Ross TJ, Hong LE, Stein EA. Individual differences in amygdala reactivity following nicotinic receptor stimulation in abstinent smokers. Neuroimage. Elsevier B.V.; 2012 Oct 27;66C:585–93.

Sweitzer, M. M., Geier, C. F., Denlinger, R., Forbes, E. E., Raiff, B. R., Dallery, J., . . . Donny, E. C. Blunted striatal response to monetary reward anticipation during smoking abstinence predicts lapse during a contingency-managed quit attempt. *Psychopharmacology 2015;* *233*(5), 751-760.

Tiffany ST, Drobes DJ The development and initial validation of a questionnaire on smoking urges. Br J Addict 1991 86:1467-1476

Toll BA, O’Malley SS, McKee SA, Salovey P, Krishnan-Sarin, S. Confirmatory factor analysis of the Minnesota Nicotine Withdrawal Scale. Psychol Addict Behav 2007;21(2):216-225.

Wang Z, Faith M, Patterson F, Tang K, Kerrin K, Wileyto EP, et al. Neural substrates of abstinence-induced cigarette cravings in chronic smokers. J Neurosci. 2007 Dec 19;27(51):14035–40.

Westbrook C, Creswell JD, Tabibnia G, Julson E, Kober H, Tindle H a. Mindful attention reduces neural and self-reported cue-induced craving in smokers. Soc Cogn Affect Neurosci. 2013 Jan;8(1):73–84.

Wingo, T.,T. Nesil,J.S. Choi.M.D. Li, Novelty Seeking and Drug Addiction in Humans and Animals: From Behavior to Molecules. Journal of Neuroimmune Pharmacology, 2016. 11(3): p. 456-470.

Yi R, Mitchell SH, Bickel WK. Delay discounting and substance abuse-dependence. In: Madden GJ, Bickel WK, editors. Impulsivity: The behavioral and neurological science of discounting. Washington, DC: American Psychological Association; 2010. pp. 191–211

Zawertailo LA, Busto U, Kaplan HL, Sellers EM. Comparative abuse liability of sertraline, alprazolam, and dextroamphetamine in humans. J Clin Psychopharmacol 1995 15(2), 117-124.

Zubieta J-K, Heitzeg MM, Xu Y, Koeppe R a, Ni L, Guthrie S, et al. Regional cerebral blood flow responses to smoking in tobacco smokers after overnight abstinence. Am J Psychiatry. American Psychiatric Association; 2005 Mar 1;162(3):567–77.

Zuckerman M, Neeb M. Sensation seeking and psychopathology. Psychiatry Research 1979; 1: 255-264.

Zuckerman M, Eysenck SB, and Eysenck HJ. (1978). Sensation seeking in England and America: Cross-cultural, age, and sex comparisons. Journal of Consulting and Clinical Psychology, 46(1), 139-149.
