# Supplementary material for: Active versus sham transcranial direct current stimulation (tDCS) as an adjunct to varenicline treatment for smoking cessation: Study protocol for a double-blind single dummy randomized controlled trial
Source: PLoS One. 2022 Dec 8;17(12):e0277408. doi: 10.1371/journal.pone.0277408 (PMC9731486; doi:10.1371/journal.pone.0277408)
Supplement: Attachment — Submitted filename: ResponsetoReviewer_secondround.docx [file pone.0277408.s007.docx]

**Reviewer**: "I only have a few last suggestions to do, to further improve the theoretical background of the present paper and its argumentation: in this regard, I would recommend deepening the information about the **application of Non-invasive brain stimulation in modulating cortical excitability, and how these techniques can be used to investigate altered brain circuits (i.e., primary sensorimotor cortex, dorsolateral prefrontal cortex and the midbrain nucleus cuneiformis) in chronic smokers**

Immune Influencers in Action: Metabolites and Enzymes of the Tryptophan-Kynurenine Metabolic Pathway

([https://urldefense.com/v3/__https://doi.org/10.3390/biomedicines9070734__;!!FxkXuJIC!fV9JcA4KFOK2ywkccMQl4FPpsWA5s2JJsDlTtOnp-ZcXs30CyE0fE9YrN_A0u189lpM-Rd9dKTNrOF1887PKWiw$](https://urldefense.com/v3/__https:/doi.org/10.3390/biomedicines9070734__;!!FxkXuJIC!fV9JcA4KFOK2ywkccMQl4FPpsWA5s2JJsDlTtOnp-ZcXs30CyE0fE9YrN_A0u189lpM-Rd9dKTNrOF1887PKWiw$) ;

The Neurobiological Correlates of Gaze Perception in Healthy Individuals and Neurologic Patients

[https://urldefense.com/v3/__https://doi.org/10.3390/biomedicines10030627__;!!FxkXuJIC!fV9JcA4KFOK2ywkccMQl4FPpsWA5s2JJsDlTtOnp-ZcXs30CyE0fE9YrN_A0u189lpM-Rd9dKTNrOF18GgbK_1A$](https://urldefense.com/v3/__https:/doi.org/10.3390/biomedicines10030627__;!!FxkXuJIC!fV9JcA4KFOK2ywkccMQl4FPpsWA5s2JJsDlTtOnp-ZcXs30CyE0fE9YrN_A0u189lpM-Rd9dKTNrOF18GgbK_1A$) ;

Functional interplay between central and autonomic nervous systems in human fear conditioning

[https://urldefense.com/v3/__https://doi.org/10.1016/j.tins.2022.04.003__;!!FxkXuJIC!fV9JcA4KFOK2ywkccMQl4FPpsWA5s2JJsDlTtOnp-ZcXs30CyE0fE9YrN_A0u189lpM-Rd9dKTNrOF18966nyuM$](https://urldefense.com/v3/__https:/doi.org/10.1016/j.tins.2022.04.003__;!!FxkXuJIC!fV9JcA4KFOK2ywkccMQl4FPpsWA5s2JJsDlTtOnp-ZcXs30CyE0fE9YrN_A0u189lpM-Rd9dKTNrOF18966nyuM$) ;

Characterizing cardiac autonomic dynamics of fear learning in humans

[https://urldefense.com/v3/__https://doi.org/10.1111/psyp.14122__;!!FxkXuJIC!fV9JcA4KFOK2ywkccMQl4FPpsWA5s2JJsDlTtOnp-ZcXs30CyE0fE9YrN_A0u189lpM-Rd9dKTNrOF18e9b52MY$](https://urldefense.com/v3/__https:/doi.org/10.1111/psyp.14122__;!!FxkXuJIC!fV9JcA4KFOK2ywkccMQl4FPpsWA5s2JJsDlTtOnp-ZcXs30CyE0fE9YrN_A0u189lpM-Rd9dKTNrOF18e9b52MY$) ;

Altered neural activity in brain cough suppression networks in cigarette smokers

[https://urldefense.com/v3/__https://doi.org/10.1183/13993003.00362-2019__;!!FxkXuJIC!fV9JcA4KFOK2ywkccMQl4FPpsWA5s2JJsDlTtOnp-ZcXs30CyE0fE9YrN_A0u189lpM-Rd9dKTNrOF18FQfQne4$](https://urldefense.com/v3/__https:/doi.org/10.1183/13993003.00362-2019__;!!FxkXuJIC!fV9JcA4KFOK2ywkccMQl4FPpsWA5s2JJsDlTtOnp-ZcXs30CyE0fE9YrN_A0u189lpM-Rd9dKTNrOF18FQfQne4$) )."

**Response**: Thank you for your comment. A paragraph has been added on p. 8 of the manuscript detailing more information on the potential of tDCS modulating cortical excitability and altered brain circuits.
